# Comparison of Vitamin D Serum Values between Rheumatoid Arthritis and Lupus Populations: An Observational Study

**DOI:** 10.2174/1874312901812010065

**Published:** 2018-04-30

**Authors:** Sahebari Maryam, Elham Atabati, Ravanshad Yalda

**Affiliations:** 1 Rheumatic Diseases Research Center, School of Medicine, Mashhad University of Medical Sciences, Mashhad, Iran; 2 Cellular and Molecular Research Center, School of Medicine, Birjand University of Medical Sciences, Birjand, Iran; 3 Clinical Research Development Unit, Ghaem Hospital, School of Medicine, Mashhad University of Medical Sciences, Mashhad, Iran

**Keywords:** Vitamin D, 25(OH) D, VitD, Rheumatoid Arthritis, Lupus, SLE, RA

## Abstract

**Background:**

In recent years, the role of Vitamin D (VitD), as an immunomedulator in autoimmune diseases, has been evaluated in basic science and practice. There is a considerable volume of data on the effect of VitD position in lupus and rheumatoid arthritis exacerbation.

**Objective:**

This study aims to compare VitD serum values in lupus (SLE) and Rheumatoid Arthritis (RA) in the geographical region of northeastern Iran.

**Methods:**

Lupus and RA Patients were selected with various disease activity levels. All the patients received an equal amount of VitD supplementation and were selected by the same inclusion and exclusion criteria. VitD serum values were measured by a commercial ELISA kit. Data were analyzed in SPSS-15.

**Results:**

A total of 148 SLE and 156 RA patients were studied. VitD serum levels were 66.54±41.2 nmol/l in the SLE group and 83.74±46.45 nmol/l in the RA group. Statistical analysis showed that VitD serum levels were lower in lupus patients than RA ones (p=0.006).

**Conclusion:**

Since VitD deficiency is very common in Iran, physiologic doses of VitD supplementation in patients lead to higher serum levels of VitD. Lower VitD values in lupus patients compared with RA ones may stem from intestinal malabsorption, higher doses of corticosteroid therapy, renal involvement and proteinuria, different polymorphisms of VitD receptors, and more sun protection strategies in lupus patients.

## 1 INTRODUCTION

Systemic Lupus Erythematosus (SLE) and Rheumatoid Arthritis (RA) are two important autoimmune diseases in which the immunomedulatory role of vitamin D (VitD) has been evaluated appropriately. However, there is still some debate about the immunomodulatory role, optimal dose, and type of VitD that should be prescribed. 

VitD has been diagnosed in recent years more as a secosteroid than a mere vitamin. It contributes to the modulation of the immune response by inhibiting actions of innate and adaptive immune cells [1]. There is some evidence that B cells are inactivated by binding VitD to VitD receptors (VDRs) on their surface [2-4]. Furthermore, VitD inhibits autoantibody production Suppression of dendritic cell-related pathways leads to the inhibition of T cell activation [5]. Additional VitD immunomodulatory activities is conducted by 1,25(OH)D [6]. VitD inhibits B cell proliferation prior to acquiring immunoglobulin secreting capabilities. It also blocks differentiation of monocytes into dendritic cells (DC), decreases the production of IL-12, INF-γ, IL-2, and enhances IL-10 production [6]. Other roles of VitD include increased monocyte-macrophage differentiation and control over macrophage responses [4-5, 7-8].

Several studies have supported the fact that VitD deficiency acts not only as a predisposing factor for RA and SLE development but also as an enhancer of disease severity and activity [2-3, 9]. On the other hand, each disease course leads to VitD deficiency due to treatment strategies, malnutrition, sedentary life style, sun protection especially in SLE, and several other factors [2-3, 9-11]. This article compares 25(OH)D serum values in patients with SLE and RA in the same geographical area by an equal amount of VitD supplementation to demonstrate differences. The hypothesis is that VitD in SLE patients may be less than in RA patients. It is important to understand the difference between RA and SLE when there is an equal dose of VitD supplementation.

## 2 MATERIALS AND METHODS

This article is a retrospective analysis of two previous cross-sectional studies in which VitD serum values in RA patients, SLE patients, and healthy populations were studied. Both studies were conducted in Mashhad, Iran, located at 360.20° latitude and 59.35° east longitude over a solar year (2013). In the two previous articles, we had a total of 148 SLE and 156 RA patients (case and control, respectively) were studied.

Over six months, from April to September, it tends to be sunny and warm in the region, if not hot. In the following six months, *i.e.* from October to March, it tends to be usually cold and cloudy [12].

A total of 99 RA patients diagnosed with ACR criteria [3] and 82 patients who fulfilled the American College of Rheumatology (ACR) criteria for lupus [2] were included for both studies in the Rheumatic Diseases Research Center affiliated with Mashhad University of Medical Sciences (2014). In both studies, the exclusion criteria included systemic co-morbidities such as chronic kidney or liver disease, malnourishment, and malabsorption (serum albumin less than 2.2 mg/dl, cholesterol below 100 mg/dl, and BMI<18.5 kg/m^2^). Smokers, pregnant women, postpartum and nursing mothers and individuals with BMI>30 kg/m^2^ were also excluded (since there is a hypothesis stating that lower VitD in overweight people may simply be representative of a larger volume of distribution of this vitamin because of fat solubility of 25(OH)D [13]). Moreover, individuals with supra-physiologic doses (>800 U/day) or injectable proportion of VitD from six months prior to sampling or those under chronic anticonvulsive therapy were excluded.

Both groups of patients received 1000 to 1200 mg calcium and 800 U VitD in addition to 400 mg hydroxychloroquine. Other medications were analyzed in detail in each article [3, 9].

VitD measurement was conducted by ELISA kit (Immundiagnostik AG, Bensheim, Germany) from the sera of participants.

Kolmogorov-Smirnov test was utilized to measure normality of data distribution. ANOVA test was used to calculate differences between VitD serum values in each group. Post-hoc analysis was used for further analysis. Advanced analysis was accomplished by linear regression model. As it was a retrospective research on previous complete data, there was no missing data.

## 3 RESULTS

A total of 82 patients (12 males, 70 females) were enrolled in the SLE group and 99 patients (11 males, 89 females) in the RA group. The average age was (29.96±11.47 yrs) in SLE and (43.44±14.31 yrs) in RA groups with a significant difference (p<0.0001). Gender distribution in the two groups was compared using chi-square test, which showed similar distribution (p=0.4). The mean duration of disease in SLE patients was 3(0.5-5.7 IQR) yrs. In RA group, disease duration was 5.9±5.6 yrs, which was significantly higher than in the SLE patients (t=0.03, Man-Whitney test).

VitD serum levels in SLE and RA groups were 66.54±41.2 nmol/l and 83.74±46.45 nmol/l, respectively. Kolmogorov-Smirnov test showed that VitD levels were not normally distributed in SLE group. Statistical analysis showed that VitD serum values were lower in SLE patients than RA ones (p=0.006, Man-Whitney test).

In this study, the values of vitD were fitted with explanatory variables (*i.e.*, age, disease duration, RA and SLE patients) and modeled by multiple linear regression technique. The final analysis is displayed in Table **1**. It shows that only lupus as a variable affects VitD serum values negatively, and age and disease duration have no statistically significant impact on VitD serum levels.

## 4 DISCUSSION

This study assessed serum values of VitD in SLE and RA patients in the northeast of Iran. We found lower plasma levels of VitD in SLE than RA under similar bio-geographical conditions, sex distribution, VitD and calcium supplementation, and the same inclusion and exclusion criteria. Regression analysis demonstrated that age did not affect VitD serum values in RA and SLE. Furthermore, regression analysis demonstrated that age and disease duration did not affect the difference in VitD serum values between RA and SLE. The mean±SD of VitD serum values in SLE patients was below optimal goal despite supplement therapy [12].

To describe the difference between RA and SLE patients, several facts are proposed. Given the important role of UVB damage in dermatological and systemic manifestations of lupus, sun protection is a rule in lupus which is considered as a risk factor for 25(OH)D deficiency [14-15].

Moreover, renal involvement in lupus may influence protein bindings for VitD and production of 1,25(OH)D. Increasing data has revealed that nephritis is a significant underlying risk factor for VitD deficiency in SLE [16].

Besides, the medication prescribed for the treatment of SLE may aggregate VitD deficiency [7]. Compared with RA, chronic higher doses of glucocorticoid therapy in SLE decline intestinal absorption and accelerate the catabolism of VitD through increasing 24α-hydroxylase activity [17-20]. Some research studies propose that the interaction between over-activation of the immune system related to T-regs in SLE can affect the activation of VitD [21-23].

Anti-VitD antibodies related to antiDNA have been evaluated in patients with SLE that may reduce VitD functions [24, 25].

Finally, polymorphism of VDR genes could be a part of VitD imbalance in SLE and RA, as different polymorphisms have been reported in those diseases [14, 26].

## CONCLUSION

This study demonstrated that 25(OH)D serum values in SLE patients are significantly lower than RA patients with the same geographic location, VitD supplementation, and other risk factors. Therefore, VitD deficiency should be considered in SLE patients more profoundly. Further studies can determine whether this is a consequence of SLE or a basic difference in the pathogenesis of two diseases.

## Figures and Tables

**Table 1:** Regression analysis on the influence of age, RA *vs.* Lupus duration and disease type on Vit D serum level as an independent variable.

Variables	P value B	B	Lower Board	Upper Board
Disease	0.044	17.45	0.44	34.45
Age	0.77	0.07	-0.45	0.61
Duration of disease	0.06	-1.17	-2.42	0.07

## DISCLOSER

We certify that there is no actual or potential conflict of interest in relation to this article.
